# Prediction and evolution of B cell epitopes of surface protein in SARS-CoV-2

**DOI:** 10.1186/s12985-020-01437-4

**Published:** 2020-10-29

**Authors:** Jerome Rumdon Lon, Yunmeng Bai, Bingxu Zhong, Fuqiang Cai, Hongli Du

**Affiliations:** School of Biology and Biological Engineering, South China University of Technology, Guangzhou, 510006 China

**Keywords:** SARS-CoV-2, Epitopes, Bioinformatics, Evolution

## Abstract

**Background:**

In order to obtain antibodies that recognize natural proteins, it is possible to predict the antigenic determinants of natural proteins, which are eventually embodied as polypeptides. The polypeptides can be coupled with corresponding vectors to stimulate the immune system to produce corresponding antibodies, which is also a simple and effective vaccine development method. The discovery of epitopes is helpful to the development of SARS-CoV-2 vaccine.

**Methods:**

The analyses were related to epitopes on 3 proteins, including spike (S), envelope (E) and membrane (M) proteins, which are located on the lipid envelope of the SARS-CoV-2. Based on the NCBI Reference Sequence: NC_045512.2, the conformational and linear B cell epitopes of the surface protein were predicted separately by various prediction methods. Furthermore, the conservation of the epitopes, the adaptability and other evolutionary characteristics were also analyzed, the sequences of the whole genome of SARS-CoV-2 were obtained from the GISAID.

**Results:**

7 epitopes were predicted, including 6 linear epitopes and 1 conformational epitope. One of the linear and one of the conformational consist of identical sequence, but represent different forms of epitopes. It is worth mentioning that all 6 identified epitopes were conserved in nearly 3500 SARS-CoV-2 genomes, showing that it is helpful to obtain stable and long-acting epitopes under the condition of high frequency of amino acid mutation, which deserved further study at the experiment level.

**Conclusion:**

The findings would facilitate the vaccine development, had the potential to be directly applied on the prevention in this disease, but also have the potential to prevent the possible threats caused by other types of coronavirus.

## Introduction

In late December 2019, a novel coronavirus was officially named as SARS-CoV-2 by the International Committee on Taxonomy of Viruses (ICTV) and identified as the pathogen causing outbreaks of SARS-like and MERS-like illness in Chinese city of Wuhan, which was a zoonotic disease. As of August 13, 2020, the outbreak of SARS-CoV-2 has been reported in many areas of the world, with more than 20,423,000 people infected [1]. With an alarming epidemicity, the reproductive number of SARS-CoV-2 has been computed to around 3.28 [2]. According to the data in the National Genomics Data Center (NGDC, https://bigd.big.ac.cn/ncov/), 15,118 genomic variations of SARS-CoV-2 has been reported at 13:00(GMT + 8) on August 13, 2020, which has aroused widespread concern.

The B cell epitope of viral surface protein can specifically bind to the host’s B cell antigen receptor and induce the body to produce protective antibody and humoral immune response. The discovery of epitopes is helpful to the development of SARS-CoV-2 vaccine and the understanding of SARS-CoV-2′s pathogenesis [3]. 3 proteins embedded in the virus envelope of SARS-CoV-2 have been identified, including spike (S), envelope (E) and membrane (M) proteins. At present, due to the lack of study of the crystal structure of surface protein of SARS-CoV-2, the study of epitopes, is time-consuming, power-consuming, costly and difficult [4], especially the conformational epitopes that depend on accurate protein structures.

In this work, we analyzed the surface protein (S, E and M protein) of SARS-CoV-2 and predicted the structures with bioinformatics methods. On this basis, we predicted the linear and conformational B cell epitopes, analyzed the conservation of the epitopes, the adaptability and other evolutionary characteristics of the surface protein, which provided a theoretical basis for the vaccine development and prevention of SARS-CoV-2. However, the results still need some experimental confirmation to ensure the validity of the application.

## Materials and methods

### Materials

All of the analyses and prediction were based on the NCBI Reference Sequence: NC_045512.2. On the basis of previous research of our group, 3624 genome sequences from GISAID (up to April 6th, 2020) were downloaded to construct a dataset for conservation analysis (Additional file 4: Table S1) [5]. The structure of S protein(PDB ID: 6 × 6p) was downloaded from RCSB PDB [6], which has a resolution of 3.22 Å [7].The data sets for S, E, and M protein were obtained by extracting the corresponding locations of the reference genome.

### Basic analysis of surface protein of SARS-CoV-2

The physical and chemical properties of target protein were analyzed by the Port-Param tool in ExPASy(Expert Protein Analysis System) [8], An online practical analysis kit for proteomics, including the primary structure of the target protein, molecular formula, theoretical isoelectric point, the protein instability index(the index < 40 means the protein was stable) and the location information. Online software, ProtScale, was used to deeply analyze the hydrophilicity and hydrophobicity of target protein and the distribution of hydrophilicity and hydrophobicity of polypeptide chains [8]. SARS-CoV-2 carried the S/E/M proteins through the virus envelope, the transmembrane region of the protein was predicted online by TMHMM 2.0 [9].

### Prediction of the 3D structure of target protein

With the amino acid sequences of the surface protein of SARS-CoV-2 of NC_045512.2 as templates, we predicted the 3D structure of E and M protein through the online server SWISS-MODEL [10] based on homology modeling method, selected the optimal structure based on the template identity and GMQE value [10], and the rationality of the structure was evaluated by Ramachandran plot[11]with PDBsum server. The structures were displayed and analyzed by SWISS-pdb Viewer v4.10 [12].

### Prediction of conformational B cell epitopes of target protein of SARS-CoV-2

Based on the structures, the conformational B cell epitopes were predicted by SEPPA 3.0 [13] and Ellipro [14] respectively, and the conformational B cell epitopes, which were predicted by all of the two methods were selected for the further analysis.

### Prediction of linear B cell epitopes of target protein of SARS-CoV-2

The Protean module of DNAStar was used to predict the flexibility [15], surface probability [16] and antigenic index [17] of the target protein of SARS-CoV-2. The linear B cell epitope was predicted by ABCpred [18] and BepiPred 2.0 [19] respectively and the common predicted linear B cell epitopes from two methods were selected for the further analysis. Coupled with the secondary structure, the tertiary structure and the glycosylation sites [20], the linear B cell epitopes were finally determined.

### Analysis of epitope conservation

Based on the PDB model and the multiple alignment result, we used the Consurf Server to analyze the conservation of amino acid sites of the epitopes online [21]. The conservation of epitopes on the surface protein of SARS-CoV-2 was analyzed by multiple alignment with MAFFT and Logo was drawn with Weblogo [22, 23].

## Results

### Basic analysis of surface protein of SARS-CoV-2

The primary structure and physicochemical properties of the S/E/M protein were analyzed. The results revealed that the S, E and M protein have average hydrophilic indexes of − 0.079, 1.128 and 0.446, respectively. On the basis of hydrophilicity, the S and M protein showed amphipathic properties, the E protein showed hydrophobic (Additional file 1: Figure S1). According to the prediction, there was an outside-in transmembrane helix in 23 residues from position 1214th to position 1236th at the N-terminal of the S protein, which was almost consistent with the study indicating that the transmembrane domain of S protein was at the position from 1213 to 1237th [24], an inside-out transmembrane helix in 23 residues from position 12th to position 34th at the N-terminal of the E protein. Two outside-in transmembrane helices of the M protein, one was in 20 residues from position 20th to position 39th, the another one was in 23 residues from position 78th to position 100th, and an inside-out transmembrane helix of the M protein in 20 residues from position 51st to position 73rd at the N-terminal, was predicted (Additional file 2: Figure S2). Each transmembrane region corresponds to a hydrophobic peak in the hydrophilic index curve. The protein instability index of the S, E and M protein were 33.01 38.68 and 39.14, which revealed that all of the S, E and M protein was stable.

### Prediction of the 3D structure of surface protein of SARS-CoV-2

**Fig. 1:**
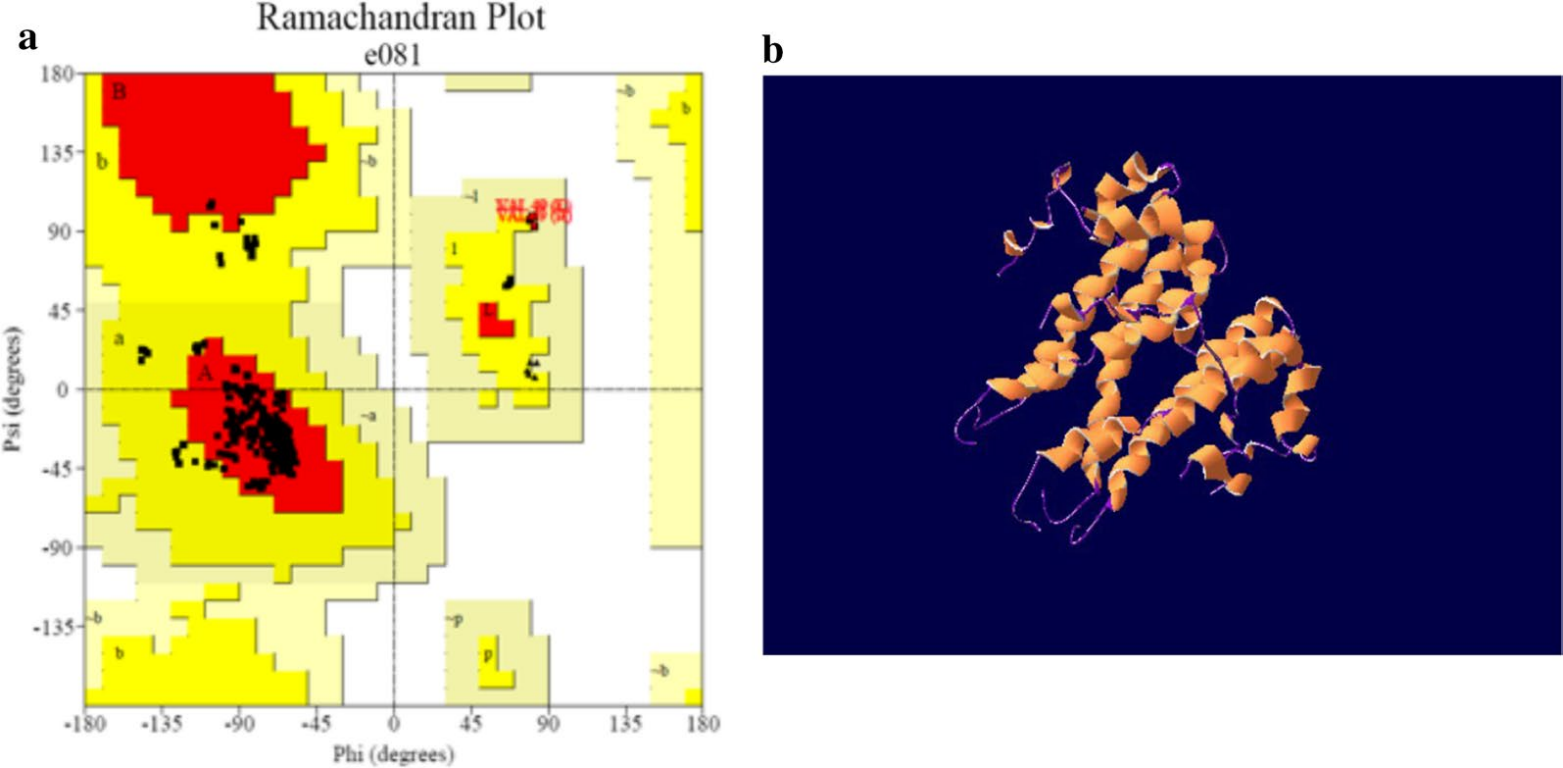
The 3D structure prediction and Ramachandran plot analysis of the E protein. **a** The Ramachandran plot analysis of the 3D structure of the E protein (without Gly and Pro). All of the residues located on the allowed region. indicating that the structure was reliable from a thermodynamic point of view. **b** The 3D structure of the E protein predicted by homology modeling. It is a pentamer with ion channel activity [38]. Its head is short, the middle of the tail is a transmembrane region which help the E protein embed in the envelope of SARS-CoV-2

The optimal template for homology modeling of the E protein of SARS-CoV-2 was the E protein of SARS (PDB ID: 5 × 29.1), with the sequence identity of 91.38% and the GMQE score of 0.73. According to the evaluation of the structure by Ramachandran plot (Fig. 1a), 100% of the residues were located in the most allowed regions (Table 1), indicating that the structure was reliable. The E protein of SARS-CoV-2 is a pentamer (Fig. 1b), which can be divided into the concentrated transmembrane part and the head located outside the envelope. The head is mainly composed of α-helix, irregular curl and turn, which is exposed to the envelope and contributes to the formation of epitopes. The tail is mainly composed of long α-helix, most of which are embedded in the envelope, hindering the formation of epitopes.

**Table 1:** The plot statistics of the Ramachandran plot

Plot statistics-E		%
Residues in most favoured regions [A, B, L]	228	84.40
Residues in additional allowed regions [a, b, l, p]	38	14.10
Residues in generously allowed regions [~ a, ~ b, ~ l, ~ p]	4	1.50
Residues in disallowed regions	0	0.00

All of the residue located on the allowed regions, which reveals that the model is reasonable on the energy level

The optimal template for homology modeling of the M protein of SARS-CoV-2 was the effector protein Zt-KP6-1(PDB ID: 6qpk. 1. A), with the sequence identity of 20.00% and the GMQE score of 0.06. The sequence identity between the optimal template and the M protein of SARS-CoV-2 and the GMQE score are too low, so that the template is not suitable for homology modeling.

### Prediction of linear B cell epitopes

**Fig. 2:**
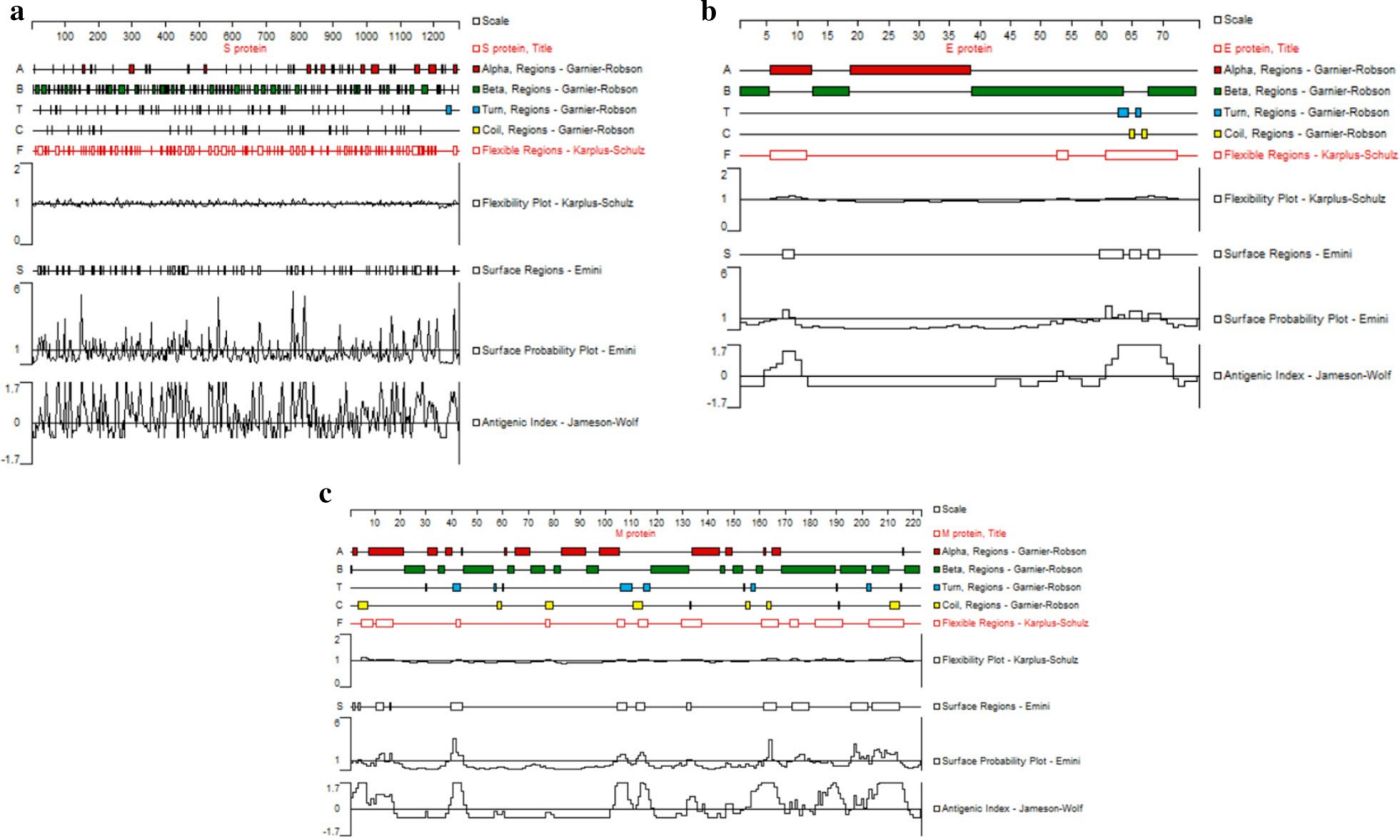
The secondary structures and properties analysis of the S, E and M protein. **a** Analysis of the S protein. It contains most α-helix and β-sheet, some Turn and Coli region, several discontinuous high flexibility fragments, fluctuant surface probability with a few of positive peak and several antigenicity regions with positive peak. The S protein showed concentrated high antigenicity peaks in 600–800 residues. **b** Analysis of the E protein. It contains most α-helix and β-sheet, some Turn and Coli region, three high flexibility fragments, few surface probability regions and two antigenicity regions with positive peak in the begin and the end of polypeptide chain, respectively. The E protein showed concentrated high antigenicity peaks in 60–70 residues. **c** Analysis of the M protein. It contains most α-helix and β-sheet, some Turn and Coli region, several high flexibility fragments, few surface probability regions, two antigenicity region with positive single peak in the begin and middle of peptide chain, respectively, and consecutive positive peaks in the end. The M protein showed concentrated high antigenicity peaks in 200–220 residues. Interestingly, the high antigenicity peaks of all three proteins were in the region where the α-helix is relatively sparse, which may be related to the fact that the α-helix structure of the helix prevents continuous residues from being located on the surface

**Fig. 3:**
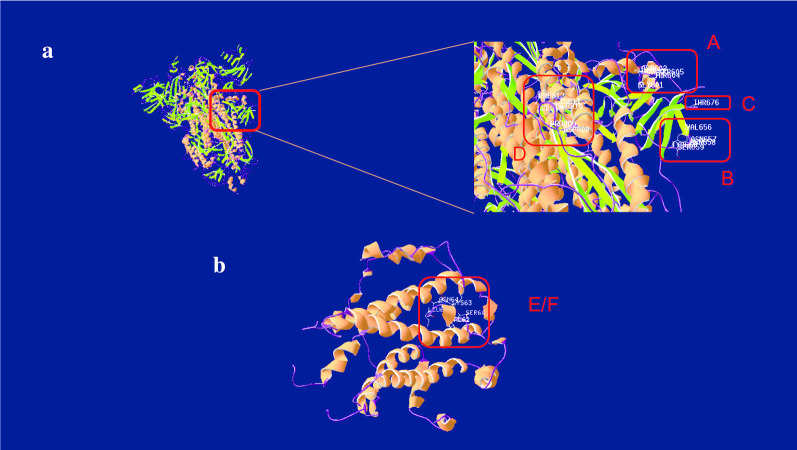
The predicted epitopes of the S and E protein. **a** The predicted linear B-cell epitopes of the S protein. The epitope A, B, C located in the forepart of the tail, the epitope D located in the back part of the tail and is close to the transmembrane region. **b** The predicted B-cell epitope of the E protein. The epitope G is the linear epitope and the F is the conformational epitope, which are coincide

All linear B cell epitopes of the surface protein were filtered according to the following criteria: (1) region with high surface probability (≥ 0.75), strong antigenicity(≥ 0) and high flexibility; (2) excluding the region with α-helix, β-sheet and glycosylation site (Fig. 2); (3) in line with the prediction by BepiPred 2.0(cut off to 0.35) and ABCpred (cut off to 0.51). Based on the results obtained with these methods and artificial optimization, we removed epitopes that are too long to be suitable for applicate, 4 potential linear B cell epitopes of the S protein were predicted (Table 2, Fig. 3a), including 601–605 aa, 656–660 aa, 676–682 aa, 808–813 aa, and they were named as the epitope A, B, C, D, respectively; 1 epitope of the E protein was selected(60–65 aa) and named as the epitope E (Table 2, Fig. 3c); 1 epitope of the M protein was selected (211–215 aa) and named as the epitope G (Table 2).

**Table 2:** The composition and the antigenic index of the epitopes of SARS-CoV-2

Name	Position	Amino acid	Antigenic index
A	601–605	GTNTS	0.525
B	656–660	VNNSY	0.575
C	676–686	TQTNSPR	0.675
D	808–813	DPSKPS	0.580
E	60–65	SRVKNL	0.588
F	60–65	SRVKNL	0.767
G	211–215	SSSSD	0.656

The scores of the epitope E and the epitope G were calculated by Ellipro, the others were calculated by Bepipred 2.0. The epitopes A, B, C and D belong to S protein, the epitopes E and F belong to E protein and they are coincident, the epitope G belongs to M protein

### Prediction of conformational B cell epitopes

With the structure of S protein (PDB ID: 6 × 6p), the conformational B cell epitopes of surface protein were predicted with Ellipro and SEPPA 3.0 with the default threshold of 0.063 and 0.5, respectively. One conformational B-cell epitope (60–65 aa) of E protein was predicted (Table 2), which is consistent with the linear epitope E. Similarly, this region located on the outside (Fig. 3c), and we selected it as a dominant conformational epitope and named F. However, the conformational epitope of the M protein could not be predicted due to the failure of credible homology modeling.

### Analysis of epitope conservation

The Consurf Server was used to predict epitope conservative sites with the structure of surface proteins and the alignment results in our dataset. Due to the lack of crystal structure of M protein, the epitope G only applies data sets to calculate conservation. In the dataset, the epitopes of S, E and M protein were basically conservative (Table 3, Additional file 3: Figure S3). Further calculation of the conservatism of the epitopes in the dataset was carried out, and the average score of all the epitopes was less than 1, which could be considered as conservative epitopes. Epitope E and epitope F from E protein had the lowest scores and showed the highest conservatism. However, it is worth noting that this value is an overall assessment of the epitopes. Residues No. 808,809 of epitope D and 214 of epitope G, as a single residue, showed a conservative score greater than 1 respectively, which revealed a risk of mutation.

**Table 3:** The conservation of the epitopes in SARS-CoV-2 dataset

Name	Position	Conservation score	Average	Name	Position	Conservation score	Average
A	601	0.482	− 0.199	E/F	60	−0.936	−0.434
	602	− 0.962			61	−0.966	
	603	−0.208			62	0.741	
	604	−0.01			63	−0.222	
	605	−0.297			64	−0.787	
					65	−0.936	
B	656	−0.332	0.114				
	657	0.243		G	211	0.086	0.282
	658	−0.341			212	0.171	
	659	0.198			213	−0.011	
	660	0.803			214	1.561	
					215	−0.395	
C	676	−0.297	0.093				
	677	0.368					
	678	−0.533					
	679	0.457					
	680	0.629					
	681	0.188					
	682	−0.159					
D	808	1.842	0.369				
	809	1.211					
	810	−0.272					
	811	−0.208					
	812	0.366					
	813	−0.723					

The calculation was independent and based on the SARS-CoV-2 data set

## Discussion

SARS-CoV-2 caused huge impact to human production, living and even life, and has become a major challenge confronting the whole world. Development of vaccine is one of the effective means of long-term prevention of the virus. Epitope vaccine is the trend of development of vaccine due to the advantages of strong pertinence, less toxic and side effects and easy to transportation and storage [25].

A group founded in March 2020 by Preston Estep, calling themselves “the Rapid Vaccine Partnership” (Radvac), has developed a very simple vaccine. In early July, Radvac published a white book detailing the vaccine they developed (https://radvac.org/). The Radvac vaccine is a "subunit" vaccine because it is composed of fragments of a pathogen, in this case it was peptide, which is essentially a short fragment of a protein that matches the SARS-CoV-2 section but does not cause disease. Subunit vaccines are already used for diseases such as hepatitis B and human papillomavirus, and a number of companies are developing subunits for COVID-19, including Novavax Biotechnology. Reliable epitopes are particularly important for the development of subunit vaccines. (https://www.technologyreview.com/2020/07/29/1005720/george-church-diy-coronavirus-vaccine/).

The determination of epitopes is the basis of the development and application of vaccine, and the clinical diagnosis. Herrera et al. [7] reported antigenic analysis of S protein obtained by ELISA, but did not study the epitopes. The conserved epitopes were predicted based on the calculation by us, which provided more reference for the immunological study of S protein. Vashi et al. predicted some epitopes based on the structure of S protein [26]. Although their studies predicted both B-cell and T-cell epitopes of S protein, they did not discuss the conservation of epitopes. We effectively supplement the study of epitopes with the conservative analysis based on a large amount of data, which can ensure the long-term effect and stability of epitopes in the application process. At the same time, their study is limited to S protein, while our study on E and M protein provides more options.

Moreover, Walls et al. [27] reported the use of conservative glycosylation sequence in S protein of SARS-CoV can stimulate neutralizing antibody against SARS-CoV-2, and the study of the Yuan et al. [28] reported that they researched the recognition of epitopes and antibodies by parsing the structure of antibody CR3022 from Rehabilitation in patients with SARS. Wang et al. [29] reported a kind of human monoclonal antibodies, which could neutralize the SARS-CoV-2, from the cell culture. What these studies have in common is that they are based on some immune responses that have already occurred. In contrast, our calculation in the computer environment is faster, but the accuracy still needs to be verified experimentally. The two methods form an effective complement.

Currently, the methods which were mainly used are X-ray scattering method, immune experiment method and bioinformatics method. The first two are time-consuming and laborious, while the bioinformatics method is gaining more and more credibility among researchers [3, 25, 30]. There are many factors to be considered in the prediction of epitopes by bioinformatics method, such as the surface probablity and flexibility of the epitopes. At the same time, it is necessary to exclude the structurally stable and non-deformable α-helix, β-sheet, glycosylation sites which may obscure the epitopes or alter the antigenicity, etc. [31]. Even so, the predicted epitopes are still inaccurate [4]. Our work takes the intersection of above methods to predict, which greatly improves the stability of the prediction. Compared with the current study on SARS-CoV-2, this work adopted various prediction methods and 3D structure databases developed in recent years, which were based on artificial neural network, Hidden Markov Model (HMM), Support Vector Machine(SVM), etc., such as ABCpred, BepiPred2.0, SEPPA 3.0, IEDB, etc. Compared with prediction by a single method [32], on the basis of a single protein [33] or on the basis of epitopes of SARS [28], these methods and databases greatly improved the accuracy of prediction and had more bioinformatic meaning. We comprehensively analyzed the prediction results from the tools which were widely used, set up screening criteria on the basis of primary structure, secondary structure and tertiary structure, so that the prediction results would more accurate and reliable.

The S protein, the E protein and the M protein are surface proteins of SARS-CoV-2 that form the outer layer of the coronavirus and protect the internal RNA, which have the potential as antigenic molecules. However, considering the current study on the epitopes prediction of SARS-CoV-2 [34] and due to the fact that S protein has been reported to be the directly binding molecule of SARS-CoV-2 to ACE2 [35], the prediction of epitopes is mainly focusing on the S protein, with few studies on the E protein and the M protein. In this work, we analyzed the S protein, the E protein and the M protein and predicted their epitopes. On this basis, 7 B cell epitopes were predicted, including 1 conformational and 6 linear B cell epitopes, one of the conformational and one of the linear are coincide. All of the epitope A, B, C, D located on the surface of the tail of the S protein, which is relatively easy to bind. The epitope E and the epitope F located at the end of the head of the E protein coincide, and this may be explained by the fact that they are all consecutive and the secondary structure avoiding the α-helix and the β-sheet. The epitope G is derived from the M protein, and the structure and conservation could not be determined due to the inability to predict reliable structure. However, it could be inferred from the surface probability scores that the epitope G is more likely to be located on the surface of the M protein.

The higher the conservation score calculated by the Consurf Server is, the more likely the site is to be mutated in the evolutionary process. When the score < 1, the site is likely to be a conservative site; when the score is between 1 and 2, the site is a site which is likely to be a relatively easy mutation; when the score > 2, the site is likely to be an easy mutation site [36]. In the 7 epitopes obtained, all the epitopes of the S, E, M protein were absolute conservative among all SARS-CoV-2 sequences. The conservation of the epitope G could not be calculated by the PDB file. Our work provides identified and conserved sites for further study. Mutations that occur during the spread of the virus can cause significant resistance to vaccine development. For example, the recently reported mutation of amino acid 614 of S protein [37] not only affects the ability of the virus to transmit, but also may affect the efficacy of vaccines involving this site. Our work provides reliable candidates for the development of epitope vaccines, but the application value of the epitopes needed further experimental verification. For example, the antigenicity of the epitope could be tested. Although the epitopes could be integrally considered to be conservative, the independent residues of these epitopes could still easy to mutate. Epitopes D and E had two and one residues, respectively, with conservative scores greater than 1, meaning that they were at risk for a single point mutation. More attention should be paid to these two epitopes in application.

The epitope detection in glycoproteins is significant to the study of the immunoreaction of SARS-CoV-2, but its challenge is less reliable than the epitope detection due to the presence of glycan [33]. In addition, SARS-CoV-2 would mutate frequently, and the epitopes predicted might mutate too, so conservative epitopes analyzed in the present study might be more reliable. According to the data from NGDC, the variation frequencies of S, E, and M proteins were 0.83, 1.02, and 0.73, respectively. Under the condition of relatively high variation frequency, the conservation of the proteins was analyzed to identify the epitopes with low mutation risk, which were important for the development of long-term and stable vaccines. However, this work is limited. Without the molecular dynamic analysis, the binding between epitopes and antibodies was not simulated to further determine the availability of epitopes, but researches from different perspectives can provide more epitopes choices for subsequent studies.

## Conclusion

In this work, we predicted 7 reliable epitopes: A, B, C, D, E/F and G. The reliability of the epitopes of the S protein was relatively better than that of the epitopes of the E protein and the M protein, indicating that the S protein is still the optimal choice for the prediction of epitopes and the development of vaccine. All of the 7 epitopes were able to achieve high conservation in SARS-CoV-2, Therefore, the epitopes not only have the potential to be directly applied on the treatment in this disease, but also have the potential to prevent the possible threats caused by other types of coronavirus. In addition, although various factors of prediction were integrated in this work, more experimental data are needed to further verify whether all the 7 epitopes can induce the body to produce corresponding antibodies and generate specific humoral immunity, due to the limited data set and other factors.

## Supplementary information

**Additional file 1**. ** Figure S1**: Deep analysis of hydrophilicity and hydrophobicity of surface protein of SARS-CoV-2. The online software, ProtScale, was used to predict the hydrophilicity and hydrophobicity of the surface protein deeply. A. The S protein has a maximum score of hydrophobicity, 3.222 at the 7th site, which revealed a strong hydrophobicity; a minimum score of hydrophobicity, -2.589 at the 679th site, which revealed a strong hydrophilicity. The score of hydrophilicity and hydrophobicity on the polypeptide chain of S protein constantly fluctuates, with most of the scores being negative, which revealed the possibility that the protein had bisexual properties on the basis of hydrophilicity. B. The E protein has a maximum score of hydrophobicity, 3.489 at the 21st and the 25th site, which revealed a strong hydrophobicity; a minimum score of hydrophobicity, -1.550 at the 65th site, which revealed a strong hydrophilicity. Most of the scores of the residues being positive, which revealed the possibility that the protein has obvious hydrophobicity. C. The M protein has a maximum score of hydrophobicity, 2.978 at the 84th site, which revealed a strong hydrophobicity; a minimum score of hydrophobicity, -1.956 at the 211th and the 212th site, which revealed a strong hydrophilicity. The scores of hydrophilicity and hydrophobicity on the polypeptide chain of M protein showed large fluctuations, and the number of positive scores and negative scores were similar, the positive scores accounted for the majority, which revealed the possibility that the protein had bisexual properties on the basis of hydrophobicity.

**Additional file 2**. ** Figure S2**: The transmembrane region of the surface protein of SARS-CoV-2. The S, E and M protein are embedded in the envelope of SARS-CoV-2, the transmembrane helix was predicted by TMHMM 2.0 server. All of three amino acid indexes were higher than 18, indicating the reliability of the prediction. A. For the S protein, an outside-in transmembrane helix was predicted in the 23 residues of amino acids from position 1214th to position 1236th at the N-terminal. The amino acid index was 23.97303. B. For the E protein, an inside-out transmembrane helix was predicted in the 23 residues of amino acids from position 12th to position 34th at the N-terminal. The amino acid index was 25.72521. C. For the M protein, 2 outside-in transmembrane helices were predicted, which were a helix in the 20 residues of amino acids from position 20th to position 39th and a helix in the 23 residues of amino acids from position 78th to position 100th at the N-terminal. An inside-out helix was predicted in the 23 residues of amino acids from position 51st to position 73rd at the N-terminal. The amino acid index was 64.90522. The calculation of the transmembrane pattern and data has clarified the position and direction of the protein in the virus, which is of great significance for the understanding of the availability of the antigen when predicting the epitopes, the epitopes located outside the virus has significant application advantages.

**Additional file 3**. ** Figure S3**: The antigenic conservation of the surface protein in SARS-CoV-2. The overall height of each stack is proportional to the sequence conservation, measured in bits, at that position, while the height of symbols within the stack indicates the relative frequency of each nucleic acid at that position. All the epitopes in the data set are highly conservative, and the serial Numbers (A-G) in the figure represent the epitopes A-G respectively.

**Additional file 4**. ** Table S1**: The list of the ID of genomes in dataset.

## Data Availability

The viral genomes described in detail here were deposited in NCBI, Genbank and GISAID.

## Abbreviations

SARS-CoV-2: Severe acute respiratory syndrome coronavirus 2
NCBI: National Center for Biotechnology Information
SARS: Severe acute respiratory syndrome
MERS: Middle east respiratory syndrome
NGDC: National Genomics Data Center
PortParam: Protein Parameters
GMQE: Global model quality estimate
PDB: Protein Data Bank
RCSB PDB: The Research Collaboratory for Structural Bioinformatics Protein Data Bank
