# Supplementary figures and images for: Prediction and evolution of B cell epitopes of surface protein in SARS-CoV-2
Source: Virol J. 2020 Oct 29;17:165. doi: 10.1186/s12985-020-01437-4 (PMC7594941; doi:10.1186/s12985-020-01437-4)

**A**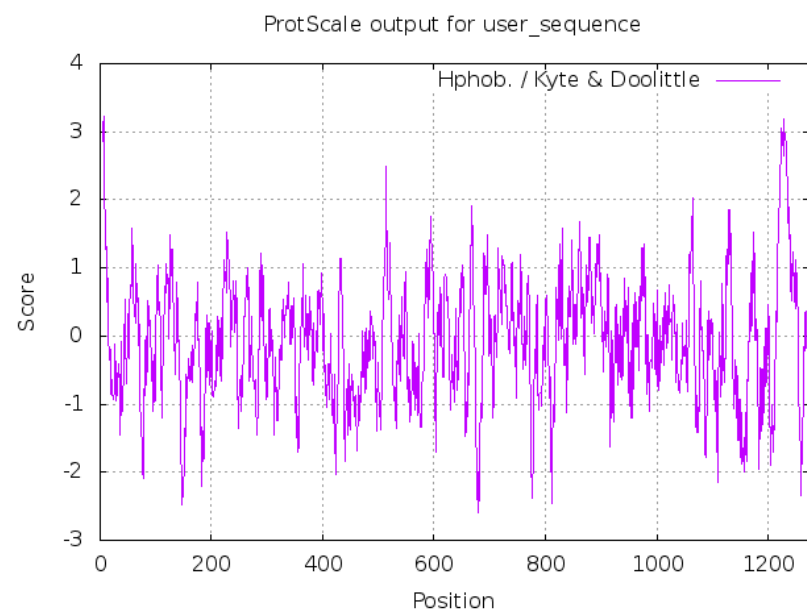**B**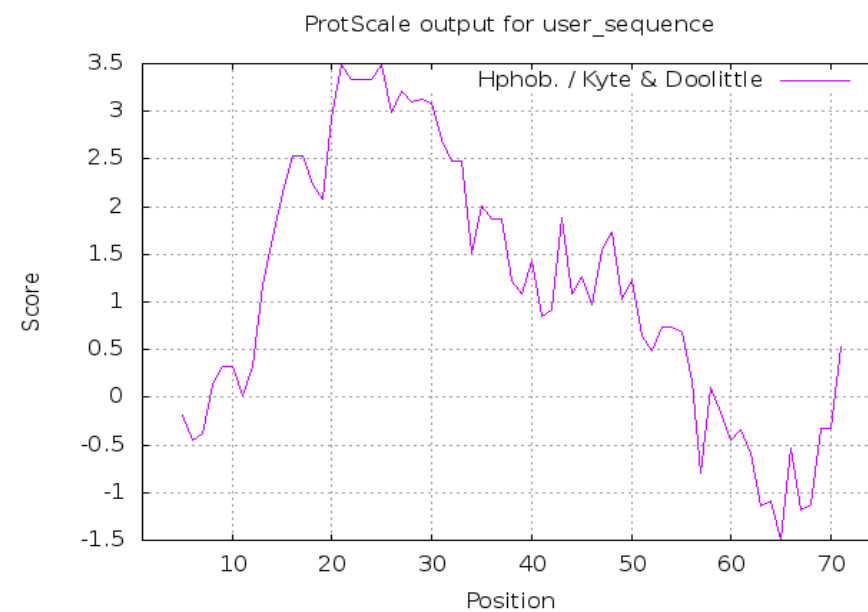**C**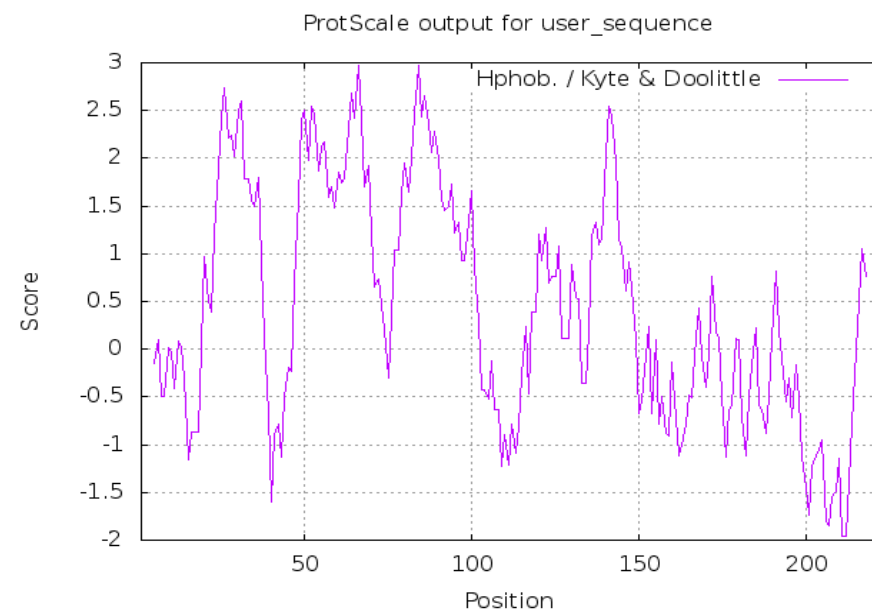

Supplement: Supplementary file 2 — Additional file 2. Figure S2: The transmembrane region of the surface protein of SARS-CoV-2. The S, E and M protein are embedded in the envelope of SARS-CoV-2, the transmembrane helix was predicted by TMHMM 2.0 server. All of three amino acid indexes were higher than 18, indicating the reliability of the prediction. A. For the S protein, an outside-in transmembrane helix was predicted in the 23 residues of amino acids from position 1214th to position 1236th at the N-terminal. The amino acid index was 23.97303. B. For the E protein, an inside-out transmembrane helix was predicted in the 23 residues of amino acids from position 12th to position 34th at the N-terminal. The amino acid index was 25.72521. C. For the M protein, 2 outside-in transmembrane helices were predicted, which were a helix in the 20 residues of amino acids from position 20th to position 39th and a helix in the 23 residues of amino acids from position 78th to position 100th at the N-terminal. An inside-out helix was predicted in the 23 residues of amino acids from position 51st to position 73rd at the N-terminal. The amino acid index was 64.90522. The calculation of the transmembrane pattern and data has clarified the position and direction of the protein in the virus, which is of great significance for the understanding of the availability of the antigen when predicting the epitopes, the epitopes located outside the virus has significant application advantages. [file 12985_2020_1437_MOESM2_ESM.pdf]

**A**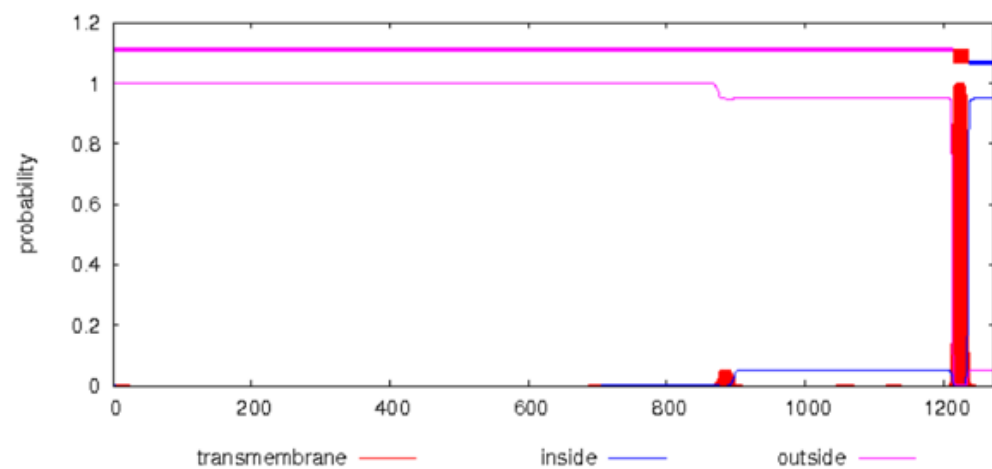**B**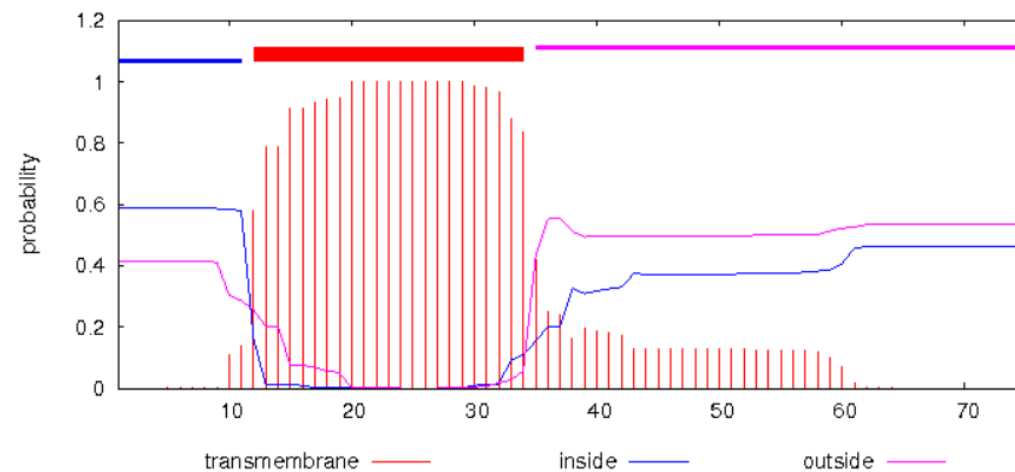**C**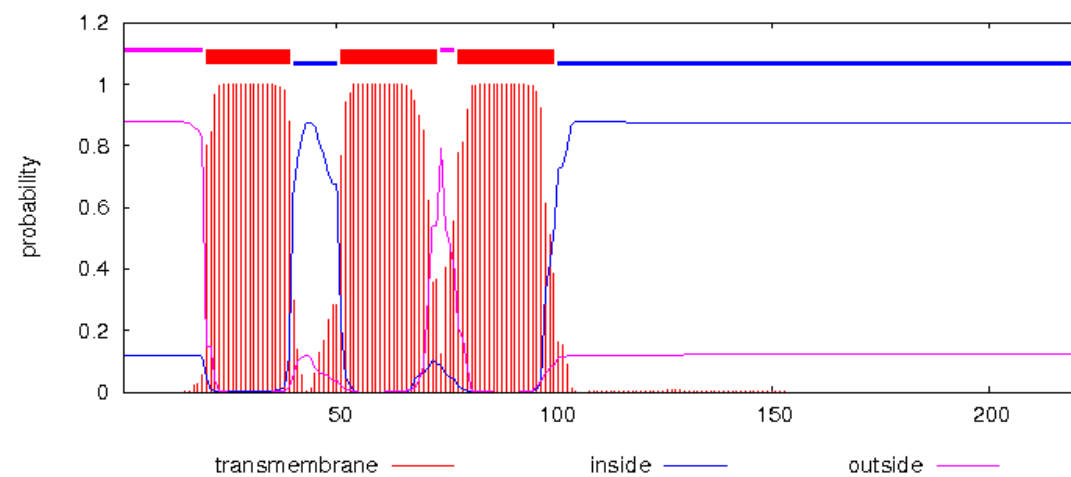

Supplement: Supplementary file 3 — Additional file 3. Figure S3: The antigenic conservation of the surface protein in SARS-CoV-2. The overall height of each stack is proportional to the sequence conservation, measured in bits, at that position, while the height of symbols within the stack indicates the relative frequency of each nucleic acid at that position. All the epitopes in the data set are highly conservative, and the serial Numbers (A-G) in the figure represent the epitopes A-G respectively. [file 12985_2020_1437_MOESM3_ESM.pdf]

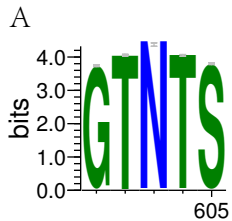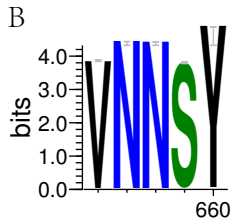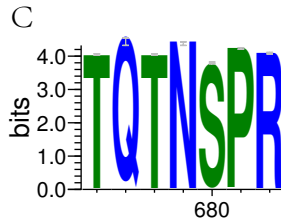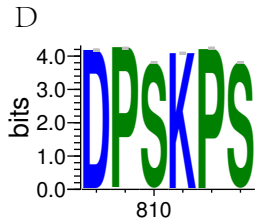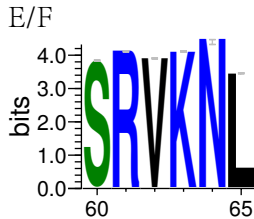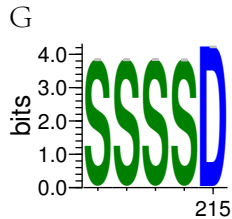

ER

no

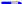

Supplement: Supplementary file 4 — Additional file 4. Table S1: The list of the ID of genomes in dataset. [file 12985_2020_1437_MOESM4_ESM.pdf]
